# Therapeutic Approaches of Ryanodine Receptor-Associated Heart Diseases

**DOI:** 10.3390/ijms23084435

**Published:** 2022-04-18

**Authors:** Norbert Szentandrássy, Zsuzsanna É. Magyar, Judit Hevesi, Tamás Bányász, Péter P. Nánási, János Almássy

**Affiliations:** 1 Department of Physiology, Faculty of Medicine, University of Debrecen, 98 Nagyerdei krt, 4032 Debrecen, Hungary; szentandrassy.norbert@med.unideb.hu (N.S.); magyar.zsuzsa@med.unideb.hu (Z.É.M.); banyasz.tamas@med.unideb.hu (T.B.); nanasi.peter@med.unideb.hu (P.P.N.); 2 Department of Basic Medical Sciences, Faculty of Dentistry, University of Debrecen, 98 Nagyerdei krt, 4032 Debrecen, Hungary; 3 Department of Orthodontics, Faculty of Dentistry, University of Debrecen, 98 Nagyerdei krt, 4032 Debrecen, Hungary; hevesi.judit@dental.unideb.hu; 4 Department of Dental Physiology and Pharmacology, Faculty of Dentistry, University of Debrecen, 98 Nagyerdei krt, 4032 Debrecen, Hungary; 5 Department of Physiology, Semmelweis University, P.O. Box 2, 1428 Budapest, Hungary

**Keywords:** ryanodine receptor, RyR, ryanopathies, congestive heart failure, catecholaminergic polymorphic ventricular tachycardia, CPVT, dantrolene, flecainide, carvedilol

## Abstract

Cardiac diseases are the leading causes of death, with a growing number of cases worldwide, posing a challenge for both healthcare and research. Therefore, the most relevant aim of cardiac research is to unravel the molecular pathomechanisms and identify new therapeutic targets. Cardiac ryanodine receptor (RyR2), the Ca^2+^ release channel of the sarcoplasmic reticulum, is believed to be a good therapeutic target in a group of certain heart diseases, collectively called cardiac ryanopathies. Ryanopathies are associated with the impaired function of the RyR, leading to heart diseases such as congestive heart failure (CHF), catecholaminergic polymorphic ventricular tachycardia (CPVT), arrhythmogenic right ventricular dysplasia type 2 (ARVD2), and calcium release deficiency syndrome (CRDS). The aim of the current review is to provide a short insight into the pathological mechanisms of ryanopathies and discuss the pharmacological approaches targeting RyR2.

## 1. Introduction

### Ca^2+^ Release in the Heart in Health and Disease

RyR2 is a ligand-gated Ca^2+^ release channel of the sarcoplasmic reticulum (SR), activated by Ca^2+^ carried by the L-type Ca^2+^ channel (LTCC) during systole. This mechanism is called Ca^2+^-induced Ca^2+^ release (CICR), which is the crucial step of cardiac excitation–contraction coupling (ECC). The Ca^2+^ current serves as an essential trigger for Ca^2+^ release, but most of the Ca^2+^ required for contraction comes from the SR. In the beginning of diastole, Ca^2+^ release is terminated and RyR2s remain closed during the whole course of diastole. [Ca^2+^]_i_ is restored by the sarcoplasmic reticulum Ca^2+^ ATP-ase (SERCA), the plasma membrane Ca^2+^ pump (PMCA), and the Na^+^-Ca^2+^ exchanger (NCX) [1].

Cardiac contractility depends on the peak systolic [Ca^2+^]_i_; therefore, it is determined by the SR Ca^2+^ content, the magnitude of the LTCC current, and the sensitivity of RyR2 to Ca^2+^ (ECC gain). Ca^2+^ fluxes through the sarcolemma, and SR are linked through a dynamic balance, which protects the cardiomyocyte against cytoplasmic Ca^2+^ overload while maintaining Ca^2+^ release amplitudes. The basis of this autoregulation is that (1) RyR2 is activated by increasing SR [Ca^2+^], (2) LTCC inactivation is Ca^2+^-dependent, and (3) NCX-mediated Ca^2+^ clearance rate is enhanced by elevated [Ca^2+^]_i_ [2,3]. The function of this mechanism was demonstrated using the RyR2 agonist caffeine [4]. The authors showed that the caffeine-induced increase of Ca^2+^ release amplitude was only transient, as the Ca^2+^ release amplitude returned to control values during treatment. In the new steady state, Ca^2+^ release operated at lower SR Ca^2+^ content but higher fractional Ca^2+^ release. The underlaying mechanism of this autoregulation was explained by the NCX activity: higher [Ca^2+^]_i_ transients drive larger Ca^2+^ efflux through the NCX, which eventually decreased the SR Ca^2+^ content to a lower steady state level. This experimental setup was designed to simulate the situation in certain heart diseases such as congestive heart failure (CHF) or catecholaminergic polymorphic ventricular tachycardia (CPVT) when RyR2 is substantially active during diastole; however, the results suggested that the autoregulation of Ca^2+^ release is broken in these conditions. Ca^2+^ leaked out of the SR and the resulting Ca^2+^ overload, which induced an inward current through the NCX, causing two problems: (1) it generated premature depolarization (delayed afterdepolarizations, DAD), associated with triggered arrythmias, and (2) depleted the SR Ca^2+^ content to severely low levels (below the capacity of autoregulation) that depressed Ca^2+^ release amplitude [5,6]. Apparently, these two points are logically inconsistent, because although low SR Ca^2+^ level explains the diminished Ca^2+^ transients and contractility in CHF, it should be associated with low arrhythmogenic propensity (since SR depletion negatively feeds back to diastolic Ca^2+^ leak). However, in contrast, Ca^2+^ leak remains paradoxically sustained in CHF, which is linked to high arrhythmogenic activity. This discrepancy might be resolved by the impaired function of Ca^2+^ handling proteins, including the RyR2 [4,7]. Next, we discuss the functional alterations of RyR that may account for the broken function of Ca^2+^ release autoregulation in heart diseases.

## 2. RyR2 Associated Heart Diseases

### 2.1. Congestive Heart Failure (CHF)

The primary causes of CHF include myocardial infarction and chronic hypertension, which initiate the morphological and functional remodeling of the cardiac tissue, resulting in cardiac hypertrophy. Cardiac remodeling is an adaptive response to increased wall stress and altered neurohormonal stimuli after myocardial infarction or hypertension, which compensates for the impaired pump function. However, in the long term, ongoing maladaptive remodeling gradually demolishes the structural integrity of the tissue, leading to decompensation and heart failure with reduced ejection fraction (HFrEF) [8,9,10,11,12]. This condition is associated with electrical instability, ventricular tachyarrhythmias, and sudden cardiac death. Although death can be prevented in many patients using implanted cardioverter-defibrillators (ICD), a large proportion of patients produce ICD-irresponsive arrhythmias. In these cases, the failure of ICDs to rescue the patient indicate that the primary cause of sudden death was acute mechanical failure, and tachyarrhythmias (if develop) are only a result of a secondary, ischemic process [13].

So far, several signaling pathways have been identified to induce hypertrophy, including the calcineurin–NFAT pathway, which is stimulated by pathological intracellular Ca^2+^ concentrations ([Ca^2+^]_i_), generated by enhanced Ca^2+^ release [14,15,16]. A critical point of this pathological signal is the impaired function of Ca^2+^ release channels, including the ryanodine receptor (RyR2-cardiac isoform), which was shown to be essential in developing hypertrophy [17,18,19,20,21,22,23,24,25]. In addition to its role in hypertrophic signaling, RyR2 is also responsible for triggered arrythmias [26,27].

#### Impaired Function of RyR2 in CHF

There is a general agreement that RyR2 is hyperactive (i.e., leaky) in CHF, and it contributes (along with reduced SERCA and enhanced NCX function) significantly to cardiac dysfunction [7,28,29]. Although the mechanism by which RyR2 becomes leaky is controversial, there is an agreement that the hyperadrenergic state in HF is an important factor [30]. The first proposal for the pathomechanism was made by Marks’ group, who showed that CHF is associated with enhanced PKA activity and increased phosphorylation state of RyR2 at the amino acid S2808 [31]. This modification would cause the dissociation of an accessory protein called FKBP12.6 (calstabin2). As FKBP12.6 stabilizes the closed state of the channel by preventing subconductive open states, RyR2 devoid of FKBP12.6 spontaneously opens during diastole, resulting in SR Ca^2+^ depletion and systolic dysfunction [31,32,33]. In line with these data, a non-phosphorylatable mutation (S2808A) prevented the progression of CHF in experimental post-myocardial infarction (MI) in mice [34]. However, other research groups failed to show high phosphorylation rates of S2808 and lower FKBP12.6 association with RyR2 and debating the role of S2808 in the regulation of RyR2 [35,36,37,38,39,40]. Moreover, Valdivia and Houser’s group showed that the S2808A mutation had no effect on the post-MI progression of CHF [41]. Furthermore, S2808A mice displayed unaltered β-adrenergic response and myocyte function, and they were not significantly protected against maladaptive cardiac remodeling [42,43]. Other studies even question the direct role of RyR2 in the regulation of cardiac contractility by PKA. For example, Bers’ group showed that PKA activation dramatically enhanced elementary Ca^2+^ release and Ca^2+^ content of the SR in wild type, but not in phospholamban-knockout cardiomyocytes, indicating that the effects of PKA on Ca^2+^ release were completely due to the phosphorylation of phospholamban, which resulted in enhanced SR Ca^2+^ load and RyR2 gating [44]. This mechanism, when luminal [Ca^2+^] reaches a threshold and triggers Ca^2+^ spillover by opening RyR2s, is called store overload-induced Ca^2+^ release (SOICR), manifested as propagating Ca^2+^ waves [45,46].

Nevertheless, PKA is not the only effector of the β-adrenergic pathway. There is solid evidence that the Ca^2+^–calmodulin-dependent protein kinase II (CaMKII) is also activated during β-adrenergic stimulation [47,48]. RyR was shown to be a CaMKII target, as CaMKII increased Ca^2+^ release in both wild-type and phospholamban-knockout myocytes [49,50]. The role of CaMKII phosphorylation on RyR2 function and RyR2-related cardiac pathology was mainly investigated using S2814A RyR2 transgenic mice in which one of the CaMKII phosphorylation sites was ablated [51]. The RyR2-S2814A mice were less prone to produce premature beats than wild-type animals [52]. Increased CaMKII activity was shown to promote HF progression by the phosphorylation of RyR2 and increased SR Ca^2+^ leak [53,54,55]. This is also supported by the result that CaMKII overexpression reduced SR Ca^2+^ load and enhanced SR Ca^2+^ release [56]. More importantly, CaMKII was found to be activated in cardiac hypertrophy and it induced dilated cardiomyopathy and HF, whereas CaMKII inhibition protected against these alterations [57,58]. In conclusion, hyperphosphorylation of RyR2 leads to increased diastolic Ca^2+^ leak in heart failure, but the effect of CaMKII is more specific on RyR2, and CaMKII phosphorylation is more important than PKA.

Another accessory protein (beside FKBP12.6), critical for the stable closed state of the channel, is calmodulin (CaM) [59]. CaM binding was shown to be low both in heart failure and some CPVT mutants, which were associated with a leaky channel phenotype [60,61]. Supporting these results, a more recent study demonstrated that enhancing the binding affinity of RyR2 to CaM by genetic modification of RyR2 reduced the expression of hypertrophy-related genes, thus suppressing the development of hypertrophy, improving intracellular Ca^2+^ signaling, and rescuing mice suffering from pressure-overload induced hypertrophy [62].

### 2.2. Catecholaminergic Polymorphic Ventricular Tachycardia (CPVT)

CPVT is an inherited disorder, linked to missense mutations of RyR2 (CPVT-1) (or other ECC proteins, including the SR Ca^2+^ buffer- and RyR-accessory protein calsequestrin (CPVT-2), CaM, Triadin, and the trans-2,3-enoyl-CoA reductase-like gene (TECRL)) [63,64]. This review discusses the most common forms of CPVT: CPVT-1 and CPVT-2, representing ≈70% and <5% of all cases, respectively. To date, more than 170 CPVT-1 linked mutations have been identified [63].

CPVT is a recognized cause of sudden cardiac death. Patients exhibit ventricular tachycardia only during catecholaminergic stimulation (e.g., during exercise or emotional stress), otherwise they are asymptomatic and present physiological ECGs at rest. Their heart is structurally normal, indicating that the excitation-contraction coupling (ECC) is intact under baseline conditions [65,66,67,68]. Moreover, normally functioning ECC implies CICR, and thus the sensitivity of RyR2 to cytoplasmic Ca^2+^ is unaltered in CPVT-1. While under catecholaminergic stimulation, when the SR Ca^2+^ content increases (due to phospholamban phosphorylation), CPVT develops. Chen’s group showed that it is the high SOICR propensity owing to low opening threshold of RyR2 to luminal [Ca^2+^] that accounts for the manifestation of CPVT (gain-of-function) [69,70]. Oversensitive RyR2s are repeatedly gated in diastole by increased SR Ca^2+^ load, which results in premature Ca^2+^ release, DADs, and tachycardia. An alternative pathomechanism is suggested by the evidence that mutant RyR2s display enhanced activity when phosphorylated by PKA. Similarly to that of CHF’s pathomechanism, Marks’s group proposed that mutant channels have lower FKBP12.6 affinity to the channels, and RyR2 phosphorylation by PKA dissociates the protein from RyR2, creating leaky channels [71]. The role of FKBP12.6 in the process, however, remains highly controversial. Many other groups reported either increased or decreased FKBP12.6 affinity for various mutants. Supporting the FKBP12.6 dissociation model, Marks’ group showed that the FKBP12.6 knockout mice were afflicted by exercise-induced sudden cardiac death, and their cardiomyocytes displayed an increased rate of DADs [71]. In contrast, Chen and colleagues did not observe CPVT phenotype (or any kind of abnormality) in mice with the same genetic background [72].

CPVT-2 is linked to the mutations of calsequestrin (CSQ). CSQ is the Ca^2+^ buffer of the SR and also a regulatory protein of RyR. CSQ was shown to inhibit RyR activity, while mutant CSQ loses control over RyR. Thus, in CPVT-2, mutant CSQ fails to suppress RyR activity, which is associated with high luminal Ca^2+^ sensitivity (i.e., low SOICR-threshold) and spontaneous diastolic Ca^2+^ release [73,74,75]. To date, 15 CPVT-2 mutations have been identified [76].

### 2.3. Calcium Release Deficiency Syndrome (CRDS)

Sudden cardiac death or aborted cardiac arrest also appear in patients carrying certain RyR2 mutations but have not shown prior symptoms or history of recurrent ventricular tachycardia. CRDS is characterized by ventricular arrhythmias, but negative CPVT. Therefore, CRDS patients produce negative results when tested with standard exercise test. However, on extended monitoring, epinephrine challenge or programmed electrical stimulation protocol with a pattern of long-burst, long-pause, and short-coupled ventricular extra-stimulus (LBLPS) provoked single ectopic events or non-sustained ventricular tachycardia (NSVT). These cardiac events could be suppressed by flecainide. Occasionally, NSVT transformed to ventricular fibrillation. Apparently, arrhythmia can be barely provoked in these patients, but when it happens, it is poorly tolerated [77].

CRDS is caused by loss-of-function mutations (or exon duplication) of RyR2, which raises the threshold of the channel for SOICR and decreases its sensitivity to both cytosolic Ca^2+^ and caffeine [77,78,79,80]. At a single cell level, early afterdepolarization (EAD) has been recorded [80]. The mechanism by which a CRDS-RyR2 could lead to ventricular tachycardia is explained by Chen’s group: when the SR load rarely reaches the higher threshold of the CRDS-RyR2, it causes EADs (known sources of re-entry), manifesting in ventricular tachycardia [78,81,82]. However, this model cannot explain why SOICR would be triggered during the terminal repolarization (just at the end of systole, in phase 2 and 3), when the SR load is the lowest during the whole heart cycle, rather than in phase 4 (diastole), when the SR is being loaded up. Therefore, we propose an alternative hypothesis for the generation of EADs, which needs to be experimentally tested. We assume that the suppressed activity of RyR would cause weaker inactivation of LTCC, resulting in prolonged Ca^2+^ influx and repolarization, with consequent activation of NCX (or reactivation of LTCC), resulting in regenerative cation current activation and EAD.

### 2.4. Arrhythmogenic Right Ventricular Dysplasia Type 2 (ARVD2)

ARVD2 (or arrhythmogenic right ventricular cardiomyopathy (ARVC)) is a cardiomyopathy linked to missense mutations of the RyR2 gene. It is characterized by thinning of the right ventricular wall, fibrofatty substitution of the myocardium, and electrical instability. ARVD2 is often responsible for the sudden cardiac deaths of juveniles and athletes. ARVD2 is clinically different from the other forms of ARVD because ARVD2 displays stress/exercise-induced ventricular arrhythmias [83,84].

These mutations are believed to destabilize the closed state of the channel, resulting in hyperactivation or hypersensitization to physiologically relevant agonists (gain-of-function). In accordance with this hypothesis, HEK293 cells overexpressing RyR2s with distinct ARVD-linked mutations showed long-lasting elevations of [Ca^2+^]_i_ following caffeine activation [85,86]. However, one of the examined mutants (L433P) exhibited lower sensitivity to activation [85,86]. Some investigators showed reduced FKBP12.6 binding to ARVD RyR2, but it was not confirmed by other groups. ARVD2 RyR2 has a lower threshold for SOICR and shows abnormal termination of Ca^2+^ release, which abnormalities are expected to exacerbate during exercise/stress and is associated with electrical instability, explaining why certain ARVD2 patients positively respond to β-blockers (including carvedilol) and flecainide [70,87,88,89,90,91].

In addition, the significant SR leak observed in the hyperactive ARVD2 phenotype should cause EC uncoupling and impaired intracellular Ca^2+^ homeostasis, which is expected to induce apoptosis/necrosis. In accordance with this, earlier reports observed apoptosis in ARVD2 [83].

### 2.5. The Role of Oxidative Stress in Cardiac Pathology

As mitochondria constitute the significant volume of cardiomyocytes, by absorbing Ca^2+^, they could act as a significant Ca^2+^ buffer to modulate Ca^2+^ signaling. However, when they get overloaded with Ca^2+^, they generate excess amounts of reactive oxygen species (ROS), which would lead to the oxidation and CaMKII phosphorylation of RyR2 and enhanced diastolic Ca^2+^ release. This pathological process is a positive feedback cycle in which leak generates further leak. Experiments using ROS probes showed that enhanced RyR2 activity (induced by caffeine or intrinsic hyperactivity in CPVT myocytes) resulted in increased mitochondrial ROS emission, oxidized RyR2, and enhanced SR leak under β-adrenergic stimulation. Importantly, the SR leak was reduced by mitochondrial ROS scavenging. Furthermore, genetic inhibition of mitochondrial Ca^2+^ uptake was associated with the reduced signal of a ROS biosensor, indicating that increased mitochondrial ROS emission depends on Ca^2+^ influx into the mitochondria [92,93].

A recent study has shown that Mito-TEMPO, a mitochondrium-targeted antioxidant with a superoxide dismutase mimetic property (but not other, non-targeted antioxidants), prevented the onset of HF or reversed established HF, preserved the contractile function and abrogated sudden cardiac death in a mouse model. These results suggest that cardiac decompensation is a mitochondrial ROS-mediated process. Therefore, recently, mitochondrial targeted antioxidants, although they have not been used clinically for the treatment of heart diseases, are considered as promising new compounds with significant therapeutic potential [94].

Earlier, redox modification of RyR2 due to the production of ROS in CHF was suggested to contribute to the enhanced RyR2 sensitivity to luminal [Ca^2+^] and also account for the development of SOICR [45,95,96,97]. SOICR is linked to increased incidence of diastolic Ca^2+^ waves, which activate an inward NCX current during diastole, resulting in DADs, the electrical events known to be the primary causes of arrythmias in failing hearts. Finally, amplified diastolic activity of RyR2 leads to attenuated systolic Ca^2+^ transients in CHF.

## 3. RyR2 as a Therapeutic Target in RyR-Related Heart Diseases

Patients hospitalized with HF have a 5-year mortality rate of 43% on average [98]. Unfortunately, the currently used antiarrhythmic drug therapy has been shown to have only little benefit on survival [99]. Additional commonly used therapeutics aim at inhibiting neurohormonal stimuli activated during the disease, such as angiotensin II, endothelin-1, TNF-α, and catecholamines. These stimuli induce hypertrophy as drivers of downstream signaling pathways. Inhibiting these hypertrophic signals using angiotensin-converting enzyme (ACE) inhibitors, angiotensin receptor blockers, aldosterone antagonists, and β-adrenergic receptor blockers are efficient drugs, as they significantly reduce the morbidity and mortality of patients. However, their therapeutic success is also limited, since the disease also continues to progress during these therapies [8,9,11].

The conventional drug therapy for CPVT combines β-blockers and Ca^2+^ channel blockers, which unfortunately only incompletely prevent sudden cardiac death [45].

Thus, there is an obvious need for new therapeutic approaches that inhibit hypertrophic signalization and prevent hypertrophy and arrhythmias. As reviewed above, both cardiac remodeling and triggered arrhythmias are Ca^2+^-dependent processes governed by diastolic SR Ca^2+^ leak, and thus therapies that prevent the functional remodeling of RyR2 are believed to be promising new strategies. Certainly, SOICR suppressors would also make a good anti-CPVT therapy. However, currently, there is not any clinically available RyR2-specific inhibitors, and the aim of this paper is to review RyR2-targeted pharmacology that has significant therapeutic potential. The summarized information about these drugs is found in Table 1.

### 3.1. Flecainide

Flecainide is a class Ic antiarrhythmic drug, a Na^+^ channel blocker. Because of its negative inotropic and proarrhythmic effects (in patients with structural heart diseases), it is contraindicated in CHF but applied in various non-structural heart diseases [100]. Flecainide showed significant efficiency in preventing ventricular arrhythmias during an exercise test in CPVT patients. The patients were recruited in the study because despite their ongoing conventional therapy (β-blocker + Ca^2+^ channel blocker), they still developed exercise-induced ventricular arrhythmias. When receiving additional flecainide therapy, 76% of them experienced partial or complete elimination of exercise-induced arrhythmias [101]. Flecainide was shown to inhibit spontaneous Ca^2+^ release events in cardiomyocytes [102,103]. The drug also inhibited RyR2 activity in single-channel current measurements, but only at positive membrane potentials that drive non-physiological ionic flux (from the cytoplasmic to the luminal side of RyR2), which questioned the direct role of RyR2 inhibition in the antiarrhythmic effect of flecainide and attributed the antiarrhythmic action entirely to Na^+^ current inhibition [104,105,106,107]. According to this alternative hypothesis, Na^+^ channel inhibition would lower the intracellular [Na^+^], resulting in higher NCX-mediated Ca^2+^ efflux, which would decrease SR Ca^2+^ content and therefore the probability of SOICR as well [103]. Very recently, Knollmann’s group resolved this debate using N-methylated flecainides [108]. They showed that the modified flecainide retained Na^+^ channel blocking ability but had reduced RyR2 inhibitory potency. The authors separated the effects attributed to Na^+^ channel or RyR2 inhibition by using the new compound as a research tool. They recorded spontaneous Ca^2+^ release events in calsequestrin-knockout cardiomyocytes (they exhibit CPVT phenotype) under experimental conditions when Na^+^ channel function was completely abolished, finding that flecainide, but not N-methylated flecainide, reduced the frequency of spontaneous Ca^2+^ release events. In addition, when the Na^+^ channels were functional, flecainide was still much more effective compared to the N-methylated version. Furthermore, flecainide, but not N-methylated flecainide, prevented ventricular tachycardia in calsequestrin-knockout mice. In these in vivo experiments, both drugs caused a similar degree of QRS prolongation, indicating that they caused equivalent Na^+^ channel block. These data suggest that RyR2 inhibition is an important component of flecainide’s antiarrhythmic action [108].

### 3.2. Carvedilol

Mortality rate in HF correlates with plasma catecholamine levels, and the therapeutic benefit of β-adrenergic receptor blockers support this finding [8,109,110]. Carvedilol is one of the most effective β-blockers that inhibit ventricular tachyarrhythmias and reduce mortality in HF patients. In addition to its β-receptor blocking effect, it also suppresses SOICR by inhibiting RyR2. There is a large difference in the carvedilol concentration required to suppress SOICR (0.3 µM) and it is necessary for the β-blocking effect (1 nM) [45]. Still, carvedilol’s SOICR-inhibiting activity may contribute to its therapeutic benefit because the drug reaches a much higher concentration in the cardiac tissue. Nevertheless, effective SOICR inhibition would probably require such higher plasma concentrations that would cause bradycardia as an adverse effect. Therefore, in order to separate these two effects, Chen’s group modified the structure of carvedilol so that it lost its β-receptor blocking effect but retained its RyR2 inhibiting action. These new compounds (VK-II-86, CS-I-34, and CS-I-59) prevented CPVT in mice but did not cause bradycardia. They also showed that VK-II-86, combined with metoprolol or bisoprolol, was more effective in suppressing ventricular tachyarrhythmias than the new drugs alone. The authors proposed that a combination therapy using selective SOICR inhibitors and β-blockers offer a promising new approach in the treatment of ventricular tachyarrhythmias [45,111].

### 3.3. Dantrolene

Dantrolene is a hydantoin derivative, indicated for use as a muscle relaxant to treat malignant hyperthermia (MH) crisis. MH is a rare idiosyncratic reaction of susceptible individuals to volatile anesthetics (e.g., halothane, isoflurane) and succinylcholine and characterized by muscle rigidity when the patient is exposed to these drugs during surgery. General skeletal muscle contracture due to high muscle work leads to the rapid increase of the body temperature, lactic acidosis, and hyperkalemia. These symptoms are fatal unless the patient is treated with the muscle relaxant dantrolene, and the body is cooled down. It turned out that MH susceptibility is linked to point mutations in the skeletal muscle-type RyR (RyR1), from which more than 200 have been discovered to date [82]. These mutations render RyR1 leaky and more sensitive to its ligands. Thus, volatile anesthetics trigger abnormal Ca^2+^ release in the resting muscle, which can be inhibited by dantrolene [112,113]. The similarity between the molecular pathomechanism of MH and cardiac ryanopathies raised the possibility that dantrolene might be an effective antiarrhythmic drug. Importantly, dantrolene did not exert adverse cardiac effects when applied in MH, and more recent studies demonstrated that the drug suppressed ventricular tachycardia [114,115]. Dantrolene (applied in 1 µM, while the therapeutic plasma concentration in MH is ≈10 µM) had antiarrhythmic effects and preserved inotropy in failing rabbit cardiomyocytes probably by inhibiting diastolic Ca^2+^ leak, increasing the threshold for SOICR and decreasing Ca^2+^ depletion from the SR. Interestingly, dantrolene remained ineffective in healthy myocytes [116]. Many other studies provided evidence that dantrolene inhibits CPVT in knock-in mice, inhibiting resting Ca^2+^ leak and spontaneous Ca^2+^ transients in myocytes derived from induced pluripotent stem cells carrying different CPVT mutations or isolated from failing human hearts. Dantrolene was also shown to suppress ventricular tachycardia in certain animal models [117,118,119,120,121]. These results raise the question as to how dantrolene acts on the two different RyR isoforms. The dantrolene binding site was identified first in RyR1. The same primary sequence in the equivalent region was found also in RyR2. Experimental data suggest that this putative binding site in RyR2 is unavailable in the healthy channel but becomes accessible for dantrolene after certain pathological modifications of the protein. It looks as though mutations and posttranslational modifications in HF disrupt certain interdomain interactions, important in stabilizing the closed state of the channel, which are believed to be re-stabilized by dantrolene [122,123]. An alternative explanation for dantrolene’s mechanism of action is that it restores CaM binding to RyR2, which provides a possible answer to the question why dantrolene affects Ca^2+^ release only in diseased hearts [60,124].

As the hepatotoxicity of dantrolene precludes its chronic therapeutic use, a safer dantrolene derivative would be favorable in therapy. The information reviewed here suggests that the development of safer and more RyR2-selective dantrolene derivatives would result in a useful, new class of antiarrhythmic drugs. Following these ideas, Laver and his colleagues discovered the effects of another hydantoin derivative, phenytoin (diphenylhydantoin), on the function of RyR2 [125]. Phenytoin is an approved drug prescribed in epilepsy. Its therapeutic benefit is mainly attributed to neuronal Na^+^ current inhibition. Moreover, it was previously shown to be antiarrhythmic [126,127]. Laver’s group found that phenytoin inhibited sheep RyR2 activity in a much lower concentration than the drug’s therapeutic plasma concentration. These effects were independent of the holding potential and CaM. However, the effect was [Ca^2+^]-dependent, as the drug was not potent at ≥10 µM cytoplasmic [Ca^2+^], indicating that it would not inhibit the amplitude of Ca^2+^ transients. Most importantly, phenytoin selectively inhibited RyR2s isolated from failing human hearts, as it did not affect RyR2s from healthy individuals [125].

### 3.4. Tetracaine Derivatives

The therapeutic benefit of RyR2 blockers observed in preclinical studies raises the question as to whether every RyR2 channel blocker would make a potentially good antiarrhythmic drug. The example of tetracaine suggests that the answer is “no”. Tetracaine was shown to abolish spontaneous Ca^2+^ release—its prolonged application caused a rebound activation of diastolic Ca^2+^ release because of the inhibition of diastolic Ca^2+^ leak resulting in elevated SR Ca^2+^ content and consequently higher SOICR propensity [102,128,129,130]. However, a similar undesirable phenomenon was not observed using flecainide, carvedilol, or dantrolene. The explanation is that while tetracaine causes long-lasting closed events (which allows for a longer time for SR overloading), flecainide and carvedilol employ a fast blocking mode of action, which only reduces the intra-burst event duration, and thus it reduces the open probability [45,102]. This mechanism will not allow SR Ca^2+^ overload because it has weaker impact on the Ca^2+^ flux through the channel. This is the reason why flecainide does not affect SR Ca^2+^ content either. In addition, flecainide was reported to lose inhibitory effects at systolic Ca^2+^ concentrations, which explains why flecainide does not decrease the amplitude of systolic Ca^2+^ release.

Recently, Abramson and his colleagues synthetized a novel tetracaine derivative, called EL20 (2-(diethylamino)ethyl 4-(butylamino)-2-methoxybenzoate), which lacks the proarrhythmic activity—at least partially—because EL20 reduces the open probability of RyR2 without inducing long-lasting closed events [131]. Interestingly, the inhibitory effect of the drug was “antagonized” by CaM. As RyR2 was demonstrated to be depleted of CaM in certain CPVTs and heart failure, EL20 may offer a selective therapeutic mechanism in CPVT and heart failure when CaM binding to RyR2 is low. Importantly, no ECG alteration was observed during EL20 treatment, indicating that Na^+^ current inhibition was not significant in the applied drug concentration during the time course of the experiment [131].

### 3.5. 1,4-Benzothiazepines

The benzothiazepine derivative K201 (4-[3(1-(4-benzyl)piperidinyl)propionyl]-7-methoxy-2,2,4,5-tetrahydro-1,4-benzothiazepine, also known as JTV519) was developed by Kirin Pharmaceutical Laboratory and first tested by Kaneko in 1994, who reported that it suppressed myocardial injury in a myofibrillar overcontraction model [132]. The author concluded that K201 acted as an “intracellular Ca^2+^ blocker”, which stimulated further experiments on the RyR2. In these studies, K201 was shown to prevent FKBP12.6 depletion from RyR2 and to restore FKBP12.6 binding to RyR2 in a canine heart failure model [133]. In another study, the drug stabilized the impaired function of the channel in vitro [134,135]. An elegant investigation by the Marks’ group used FKBP12.6 knockout (FKBP−/−) and haploinsufficient (FKBP+/−) mice to test the role of FKBP12.6 in the drug’s antiarrhythmic action. Both FKBP−/− and +/− mice exhibited exercise-induced ventricular tachycardia, but only the FKBP+/− mice were rescued from sudden cardiac death by K201 treatment. They also provided single-channel current data to show that the open probability of RyR2 isolated from exercised FKBP+/− mice was significantly lower in the case of K201-treated animals, but this inhibitory effect was not observed on RyR2s isolated from the FKBP−/− mice. In further experiments, cardiac SR vesicles of wild-type mice were subject to PKA treatment, which was supposed to strip FKBP off RyR2, and these channels were incorporated into planar lipid bilayers. FKBP12.6 was added to the channels, but this addition remained without effect, unless K201 was also included into the medium. These data strongly support the idea that the pharmacological effect of the drug is linked to FKBP12.6 binding to RyR2. Furthermore, a binding assay demonstrated that K201 increased the affinity of RyR2 for FKBP12.6 [136]. In line with these data, K201 was also shown to stabilize CPVT RyR2s in a closed state by enhancing FKBP12.6 binding [137,138].

In order to determine whether restoring FKBP12.6 binding to RyR2 improves cardiac function in CHF, MI was induced in wild-type and FKBP−/− mice. K201-treated wild-type mice showed a significant increase in ejection fraction, while K201 failed to cause improvement in FKBP−/− mice compared to placebo controls. Coimmunoprecipitation experiments demonstrated an increased amount of FKBP12.6 bound to RyR2 in K201-treated animals. These data suggest that FKBP12.6 is essential for the beneficial effects of K201 in CHF, and K201 acts by enhancing FKBP12.6 affinity to RyR2 [139].

K201 has non-specific Na^+^, Ca^2+^, and K^+^ current inhibiting actions that motivated further research to develop newer derivatives (collectively called rycals) that lack these off-target effects. One compound from this group, S107, was reported to have K201-like effects [33,140,141]. S107 inhibited the SR Ca^2+^ leak and reduced ventricular arrhythmias, infarct size, and left ventricular remodeling in a rat ischemia–reperfusion model [142]. Clinical trials using rycals were scheduled to begin in 2009 and have shown promising results in pilot clinical trials for the treatment of heart failure and cardiac arrhythmias.

## 4. Conclusions

Fixing dysregulated Ca^2+^ release offers an effective therapy in both CHF and CPVT; however, currently, there is not any clinically available RyR2-specific inhibitor. There is a substantial effort to find RyR2-selective inhibitors, but not all RyR2 blockers would theoretically make an appropriate antiarrhythmic medicine. Drug repurposing looks to be the primary strategy in RyR2-targeted drug development and modification of these lead molecules have promising prospects. These efforts probably will lead to many newer RyR2-selective drugs with strong therapeutic potential soon.

## Figures and Tables

**Table 1:** RyR2 inhibitors used in ryanopathies.

Ryanopathy	Name	Original Use	RyR-Specific Derivative	Status of Trial
Congestive Heart Failure (CHF)	Carvedilol	Beta-blocker		clinical
			VK-II-86	preclinical
	Dantrolene	RyR1		preclinical
	Phenytoin	Na^+^ channel		preclinical
	K201 (JTV519)	RyR2		preclinical
Catecholaminergic Polymorphic Ventricular Tachycardia (CPVT)	Carvedilol			clinical
			VK-II-86	preclinical
	Flecainide	Na^+^ channel		clinical
	Dantrolene			preclinical
	(Tetracaine)		EL20	preclinical
	K201 (JTV519)			preclinical
Calcium Release Deficiency Syndrome (CRDS)	Flecainide			clinical
			N-methylated	preclinical
Arrhythmogenic Right Ventricular Dysplasia type 2 (ARVD2)	Carvedilol			clinical
	Flecainide			clinical
